# Native mass spectrometry prescreening of G protein-coupled receptor complexes for cryo-EM structure determination

**DOI:** 10.1063/4.0000806

**Published:** 2025-10-27

**Authors:** Donggyun Kim, Weijing Liu, Vadim Cherezov, Rosa I Viner

**Affiliations:** 1 University of Southern California, Los Angeles, CA, USA; 2 Thermo Fisher Scientific, San Jose, CA, USA

## Abstract

G protein-coupled receptors (GPCRs) are essential transmembrane proteins playing key roles in human health and disease. Understanding their atomic-level molecular structure and conformational states is imperative for advancing drug development. Recent breakthroughs in single-particle cryo-EM have propelled the structural biology of GPCRs into a new era. Nevertheless, the preparation of suitable GPCR samples and their complexes for cryo-EM analysis remains challenging due to their poor stability and highly dynamic nature. Here, we present our online buffer exchange-native MS method combined with Direct Mass Technology (OBE-nMS+DMT) which facilitates high-throughput analysis and guides sample preparation. We applied this method to optimize the GPR119-Gs complex sample prior to cryo-EM analysis, leading to a 3.51 Å resolution structure from only 396 movies collected on a 200 kV Glacios. This study suggests that the OBE-nMS+DMT method emerges as a powerful tool for prescreening sample conditions in cryo-EM studies of GPCRs and other membrane protein complexes.

